# Virulence and immunogenicity of genetically defined human and porcine isolates of *M*. *avium* subsp. *hominissuis* in an experimental mouse infection

**DOI:** 10.1371/journal.pone.0171895

**Published:** 2017-02-09

**Authors:** Nicolas Bruffaerts, Christelle Vluggen, Virginie Roupie, Lucille Duytschaever, Christophe Van den Poel, Joseph Denoël, Ruddy Wattiez, Jean-Jacques Letesson, David Fretin, Leen Rigouts, Ophélie Chapeira, Vanessa Mathys, Claude Saegerman, Kris Huygen

**Affiliations:** 1 Service Immunology, Operational Direction Communicable and infectious Diseases, Scientific Institute of Public Health, Brussels, Belgium; 2 Service Bacterial diseases, Operational Direction Communicable and infectious Diseases, Scientific Institute of Public Health, Brussels, Belgium; 3 Unit Bacterial Zoonoses of livestock, Operational Direction Bacterial Diseases, Veterinary and Agrochemical Research Centre, Brussels, Belgium; 4 Research Unit in Epidemiology and Risk Analysis applied to Veterinary Sciences, Fundamental and Applied Research for Animal and Health, Université of Liège, Liège, Belgium; 5 Service Protéomique et Microbiologie, Université de Mons, Mons, Belgium; 6 Unité de Recherche en Biologie des Microorganismes, Université de Namur, Namur, Belgium; 7 Department Biomedical Sciences, University of Antwerp, Antwerp, Belgium; 8 Unit Mycobacteriology, Institute of Tropical Medicine, Antwerp, Belgium; 9 Service bioinformatique, Genoscreen, Lille, France; Institut National de la Recherche Agronomique, FRANCE

## Abstract

*Mycobacterium avium* subsp. *hominissuis* (*Mah*) represents a health concern for humans and to a lesser extent for pigs, but its zoonotic potential remains elusive. Using multispacer sequence typing (MST) we previously identified 49 different genotypes of *Mah* among Belgian clinical and porcine isolates, with 5 MSTs shared by both hosts. Using experimental intranasal infection of BALB/c mice, we compared the virulence and immunogenicity of porcine and clinical human isolates with shared genotype or with a genotype only found in humans or pigs. Bacterial replication was monitored for 20 weeks in lungs, spleen and liver and mycobacteria specific spleen cell IFN-γ, IL-10 and IL-17 production as well as serum antibody responses were analyzed. Isolates varied in virulence, with human and porcine isolates sharing MST**22** genotype showing a thousand fold higher bacterial replication in lungs and more dissemination to spleen and liver than the human and porcine MST**91** isolates. Virulent MST**22** type was also associated with progressive suppression of IFN-γ and IL-17 responses, and increased IL-10 production. Whole genome sequencing of the two virulent isolates with MST**22** genotype and two avirulent isolates of genotype MST**91** and comparison with two well-studied *M*. *avium* subsp. *hominissuis* reference strains i.e. *Mah* 104 and *Mah* TH135, identified in the two MST**22** isolates nine specific virulence factors of the mammalian cell entry family, that were identical with *Mah* 104 strain. Despite the obvious limitations of the mouse model, a striking link of virulence and identity at the genome level of porcine and human isolates with the same multisequence type, for which no correlation of place of residence (humans) or farm of origin (pigs) was observed, seems to point to the existence in the environment of certain genotypes of *Mah* which may be more infectious both for humans and pigs than other genotypes.

## Introduction

Among non-tuberculous mycobacteria (NTM), bacteria of the *Mycobacterium avium* complex (MAC) are the most frequently isolated from patients [1,2]. The *M*. *avium* species is divided into four subspecies: *M*. *avium* subsp. *avium* (*Maa*), *M*. *avium* subsp. *silvaticum* (*Mas*), *M*. *avium* subsp. *paratuberculosis* (*Map*) and *M*. *avium* subsp. *hominissuis* (*Mah*) [3,4]. Recently, a phylogenetic study showed that *Mah* represents diverse groups of organisms from which two distinct groups, *Map* and *Maa/Mas*, have evolved independently [5]. These four subspecies of *M*. *avium* are genetically close but they differ widely in their host range and pathogenicity. Indeed, *Map* is responsible for an intestinal illness in ruminants known as Johne’s disease [6] and could be implicated in human Crohn’s disease [7]. *Maa* and *Mas* mainly infect birds causing a tuberculosis-like disease, whereas *Mah* is a frequent agent of human and pig mycobacterioses [8–10]. As indicated by its name, *M*. *avium* subsp. *hominissuis* is the most frequently recovered *M*. *avium* subspecies in swine, although *Maa* can also be found. Although both subspecies can infect pigs, Agdestein *et al*. observed more extensive shedding of *Mah* than of *Maa*, which might explain the higher incidence of infection caused by the former subspecies [9,11].

In humans, *Mah* is the causative pathogen of two main types of disease: disseminated disease in immunocompromised hosts, such as AIDS patients and pulmonary disease in individuals without systemic immunosuppression [12]. Moreover, an association between *Mah* and human lymphadenitis has been described by Despierres *et al*. [13]. In a retrospective study of 67 MAC-infected patients diagnosed between July 1996 and March 2011, *Mah* was isolated from 24 out of 25 lymphadenitis patients, who were significantly younger than 42 non-lymphadenitis patients. Also, cervical topography found in 76.5% of lymphadenitis patients was significantly more frequent in non-immunocompromised patients (p = 0.04) [14]. In the French study, multispacer sequence typing (MST) of *Mah* isolates revealed an enormous genetic variability with 15 genotypes in 29 non-lymphadenitis isolates (molecular diversity, 0.622) versus 11 genotypes in 24 lymphadenitis isolates (molecular diversity, 0.578) [13]. Using the same multispacer sequence typing method as described by Despierres *et al*., we previously identified 49 different genotypes of Belgian *Mah* among pig isolates collected during 2012–2013 (11 MST types) and among clinical human isolates collected during 2011–2012 (43 MST types), and we observed that only 5 of these MST genotypes were shared by both hosts [15]. MST**12** and MST**22** genotypes were frequently found both in human and in pigs, whereas MST**15**, the second most frequently observed type in humans, was not detected in pigs. In the Despierres *et al*. study, 12/67 clinical isolates were typed as MST**13** whereas in our study only 2/92 clinical isolates had that genotype [13]. In contrast, in France only 1/67 isolates had the MST**22** genotype (isolated from a 40 years old male patient with pulmonary disease) whereas in our study MST**22** genotype was found in 5/92 clinical isolates (and in 7/54 porcine isolates). Finally, MST**12** genotype was not detected in the French study, whereas in Belgium this was really the preponderant genotype (16/92 human and 28/54 porcine isolates). In order to determine the evolution of *M*. *avium* infecting pigs over time, we recently performed a subspecies determination and genotyping on two older panels of porcine isolates (collected in 1967–1968 and 1992–1996). Unlike the omnipresence of *Mah* reported among the 2012–2013 isolates, a significant presence of both *Maa* and *Mah*, was observed in these older panels. Multispacer sequence types (MST) changed over time, although MST**12** was already the predominant genotype in the older *Mah* panels as well (Soetaert *et al*., submitted for publication).

As *Mah* represents an increasing public health concern given its pathogenicity for humans, a detailed comparison of human clinical isolates and swine isolates could contribute to establish or exclude any epidemiological link between both hosts. Here we report on a comparison of bacterial replication and immunogenicity of *Mah* isolates with defined MST type in an experimental mouse model.

Since *Mah* has been recovered from the environment, more particularly in sawdust and peat [16], an intranasal infection was used to mimic airway exposure. We have previously shown that the BALB/c mouse strain shows the same susceptibility to *M*. *avium* subsp. *paratuberculosis* infection as the mutant C57BL/6 ^*bg/bg*^ mouse strain (considered to be the best mouse model for *Mycobacterium avium* complex disease [17]) and can be used for assessing mycobacterial virulence by monitoring bacterial replication in spleen, lungs and liver, as well as for immunogenicity studies [18].

BALB/c mice were infected by the intranasal route with four human and four porcine *Mah* isolates, with a shared MST genotype or with an MST genotype only found in humans or pigs. Bacterial replication was monitored for 20 weeks in lungs, spleen and liver and mycobacteria specific spleen cell IFN-γ, IL-10 and IL-17 production as well as serum antibody responses were analyzed.

For a porcine and human isolate with shared MST**22** genotype and for a porcine and human isolate with shared MST**91** genotype, we previously described the full genome sequencing [19], and in this paper we compared these sequences to those of two well-studied *M*. *avium* subsp. *hominissuis* reference strains i.e. *Mah* 104 [20] and *Mah* TH135 [21].

## Materials and methods

### Ethics statement

Clinical isolates of *Mah* were used with the approval of the ethical committee of Hôpital Erasme (ULB) (reference P2014/028). The study protocol was approved by the Ethical Committee of the Centrum voor Onderzoek in Diergeneeskunde en Agrochemie-Pasteur Instituut van Brussel-Wetenschappelijk Instituut voor Volksgezondheid regulations (CODA-PIB-WIV, permit number: 060202–02, President Dr. Els Goossens).

### Clinical and porcine isolates

Table 1 shows the list of the eight *Mah* isolates used in this study. The four clinical human isolates of *Mah* were isolated from patients diagnosed by the Tuberculosis & Mycobacteria National Reference Laboratory of the Scientific Institute of Public Health (WIV-ISP) in Belgium in 2012 and the last trimester of 2011. Porcine *Mah* isolates were isolated from submandibular lymph nodes with visible lesions (identified after veterinary inspection in 2012–2013 by the UREAR-ULg) by the Belgian veterinary centre CODA-CERVA. The different porcine *Mah* isolates originated from different farms, and farm location had no relation with the selected human isolates [15].

**Table 1:** List of the porcine and human *M*. *avium* subsp. *hominissuis* isolates used in this study.

**Human clinical *Mah* isolates**					
Isolate name	MST code	Ref. name in [19]	Age	Sex	Symptoms
Hu**12**	12	12_009	Unknown	Female	Unknown
Hu**15**	15	12MY0204	4 years	Female	Lymphadenitis
Hu**22**	22	12_062	40 years	Male	HIV neg, disseminated
Hu**91**	91	12_067	40 years	Male	AIDS, disseminated
**Porcine *Mah* isolates**					
Isolate name	MST code	Ref. name in [19]	Symptoms		
Po**12**	12	LYM108	Mandibular lymphadenitis		
Po**22**	22	LYM122	Mandibular lymphadenitis		
Po**91**	91	LYM86	Mandibular lymphadenitis		
Po**103**	103	LYM119	Mandibular lymphadenitis		

### *Mah* identification and MST typing

*M*. *avium* species identification was realized as a routine activity of the reference laboratory, by sequencing of the gene coding for the 16SrRNA, as previously described [22]. All the *Mah* isolates were identified at the subspecies level by sequencing of the PCR-amplified *rpoB* gene and by IS*1245*/IS*901* analysis. Genotyping of the *Mah* isolates was performed by Multispacer Sequence Typing MST. The PCR amplification of the spacers MST2, MST4, MST15 and MST16 was realized as described by Cayrou *et al*. [23]. Purification of the amplicons and sequencing was realized as described above, with the same forward and reverse primers as used for the different MST PCRs [23]. The MST type was determined by consultation of the MST database of the “Université de la Méditerranée” accessible via the following link: http://ifr48.timone.univ-mrs.fr/mst/mycobacterium_avium/

In total 49 different genotypes of *Mah* were identified among pigs (11 MST types among 54 isolates) and humans (43 MST types among 92 isolates), with only 5 MST types shared by both hosts [15]. As we have previously indicated, no correlation was observed between MST type and place of residence or the farm of origin for human and porcine isolates respectively [15].

### *Mah* cultivation

The *Mah* isolates were grown in plastic Roux flasks in liquid Middlebrook 7H9 medium (BD, Franklin Lakes, NJ, USA), containing 10% Oleic Acid-Albumin-Dextrose-Catalase, for 14 days at 39°C under static aerobic conditions in horizontal position to maximize oxygen supply. Bacteria were harvested by centrifugation, washed three times with PBS and then frozen at -80°C in Middlebrook 7H9 medium supplemented with 20% glycerol until use. After one freeze/thaw cycle and 2 min sonication, stocks were enumerated by plating serial dilutions on Middlebrook 7H11 (BD, Franklin Lakes, NJ, USA) supplemented with 10% OADC. The list of the eight *Mah* isolates used for infection is given in Table 1.

### Whole genome sequencing

Whole genome sequencing was performed on the human and porcine MST**22** isolates and on the human and porcine MST**91** isolates, with an ILLUMINA Miseq 2X150 bp and a quality analysis was realized using FastQC v0.11.5. Total genome sequence length and number of protein coding sequences were previously reported and whole sequences have been deposited at the European Nucleotide Archive [19]. For the sake of clarity, the 12_062, 12_067, LYM122 and LYM086 isolates have been renamed here as Hu**22**, Hu**91**, Po**22** and Po**91** respectively.

### Mice

Female BALB/c mice aged 6–8 weeks were bred and kept (5 animals per standard cage) at the WIV-ISP experimental animal facilities (Site Ukkel), complying with the Belgian legislation that transposes European Directive 2009/41/EC, repealing Directive 90/219/EC (EC, 2009). All efforts were made to minimize animal suffering. Anaesthesia was performed using isoflurane.

### Experimental infection

Mice were infected by the intranasal route with 10^7^ CFU of *Mah*. Infection dose was verified by plating serial dilutions on solid Middlebrook 7H11 agar supplemented with 10% OADC. After blood sampling, animals (n = 5/group at each time point) were sacrificed by cervical dislocation. Two infection runs were realized, one run with the eight isolates and mice sacrificed at week 1, 5, 10 and 20 weeks post-infection and a second run with seven of the eight isolates (Hu**15** isolate missing), and with time-point measures at 5, 9 and 20 weeks post-infection. Non-infected mice were used as negative control. Spleen, lungs and liver of individual animals were removed aseptically. Bacteria were enumerated by plating serial threefold organ dilutions in duplicate on solid Middlebrook 7H11 agar supplemented with 10% OADC. Petri dishes were incubated for 14 days at 39°C and number of colonies were counted visually.

### Cytokine production

Spleens were collected at week 5 and week 20 post-infection and homogenized using a loosely fitting Dounce homogenizer. Leucocytes (4 x 10^6^ cells/mL as measured in a Coulter Counter) were cultivated at 37°C in a humidified CO_2_ incubator in round-bottom microwell plates in a volume of 200 μL RPMI-1640 medium (Life Technologies, Carlsbad, CA, USA) supplemented with 5 x 10^−5^ M 2-mercaptoethanol, 1% penicillin-streptomycin and 10% fetal calf serum (FCS). Spleen cells of five mice per group were analysed individually. Cells were stimulated with Culture Filtrate (CF) of *Map* [24] or with purified recombinant *E*. *coli*-expressed *Map* proteins at a final concentration of 5 μg/mL [24,25]. Culture supernatants were harvested after 72h. Supernatants were stored frozen at -20°C until analysis.

### Cytokine ELISA

IFN-γ, IL-10 and IL-17A were quantified in 72h culture supernatants of spleen cell cultures using a sandwich enzyme-linked immunosorbent assay (ELISA). IFN-γ was detected using coating antibody R4-6A2 and biotinylated detection antibody XMG1.2 (BD Pharmingen, Franklin Lakes, NJ, USA). Plates were revealed with O-phenylenediamine dihydrochloride substrate (OPD; Sigma-Aldrich, St. Louis, MO, USA); the reaction was stopped with 1 M H_2_SO_4_, and the optical density was read at 490 nm. IL-10 and IL-17A were detected with commercial ELISA kits (eBioscience, San Diego, CA, USA).

### Characteristics of 35 recombinant *Map* proteins

The list of 35 *Map* proteins is given in Table 2. Antigens 1–18 were previously identified by proteomic and immunoproteomic analysis of standard *Map* biofilm culture filtrate of bacteria grown as a surface pellicle on synthetic Sauton medium [26]. Antigens 19–24 were identified using an *in silico* analysis of *Map* genome [27]. Finally, antigens 25–35 were identified by proteomic analysis of in Sauton culture filtrate of *Map* submitted to different stress conditions (hypoxia, acidic pH, nutrient starvation or non-toxic NO) as described for *M*. *tuberculosis* by Voskuil *et al*. [28]. In total, seven proteins were found to be up-regulated under stress conditions (highlighted in grey) (Roupie *et al*., manuscript in preparation). The 35 proteins were produced as recombinant histidine-tagged proteins in *E*. *coli* and purified by affinity chromatography on immobilized nickel-chelate (Ni-NTA) columns.

**Table 2:** List of 35 recombinant *M*. *avium* subsp. *paratuberculosis* antigens.

**Nr.**	**Antigen**	Description	E value	Ident.	Accession	Myco
1	**MAP0139c**	transcriptional regulator, PadR family protein [Mycobacterium avium 104]	8,00E-144	99%	ABK65696.1	**MAA104**
2	**MAP0586c**	transglycosylase SLT domain protein [Mycobacterium avium 104]	0	99%	ABK65241.1	**MAA104**
3	**MAP0907**	morphine 6-dehydrogenase [Mycobacterium avium 104]	0	98%	ABK68334.1	**MAA104**
	**MAP0907**	oxidoreductase [Mycobacterium avium subsp. hominissuis TH135]	1,00E-52	41%	BAN32966.1	**MAH TH135**
4	**MAP1438c**	alpha/beta hydrolase fold domain protein [Mycobacterium avium 104]	0	99%	ABK65668.1	**MAA104**
	**MAP1438c**	alpha/beta hydrolase [Mycobacterium avium subsp. hominissuis TH135]	2,00E-24	40%	BAN33440.1	**MAH TH135**
5	**MAP1562c**	conserved hypothetical protein [Mycobacterium avium 104]	1,00E-89	100%	ABK66813.1	**MAA104**
6	**MAP1693c-FL**	peptidyl-prolyl cis-trans isomerase, fkbp-type domain protein [Mycobacterium avium 104]	1,00E-125	98%	ABK64725.1	**MAA104**
	**MAP1693c-FL**	peptidyl-prolyl cis-trans isomerase domain-containing protein [Mycobacterium avium subsp. hominissuis TH135]	4,00E-113	100%	BAN31629.1	**MAH TH135**
7	**MAP2411**	pyridoxamine 5-phosphate oxidase [Mycobacterium avium subsp. hominissuis 10–4249]	9,00E-101	100%	ETB30907.1	**MAH**
8	**MAP2677c**	glyoxalase family protein [Mycobacterium avium 104]	8,00E-92	98%	ABK69112.1	**MAA104**
9	**MAP2746**	hypothetical protein MAH_1788 [Mycobacterium avium subsp. hominissuis TH135]	4,00E-119	98%	BAN30862.1	**MAH TH135**
	**MAP2746**	conserved hypothetical protein [Mycobacterium avium 104]	2,00E-109	99%	ABK64828.1	**MAA104**
10	**MAP-CF036-Ag3**	integral membrane protein YrbE1A [Mycobacterium avium subsp. hominissuis TH135]	3,4	20%	BAN33502.1	**MAH TH135**
	**MAP-CF036-Ag3**	conserved hypothetical protein [Mycobacterium avium 104]	3,4	20%	ABK66937.1	**MAA104**
11	**MAP3385**	methyltransferase [Mycobacterium avium subsp. hominissuis TH135]	8,00E-65	64%	BAN32924.1	**MAH TH135**
	**MAP3385**	hypothetical protein MAH_3962 [Mycobacterium avium subsp. hominissuis TH135]	2,00E-64	44%	BAN33036.1	**MAH TH135**
	**MAP3385**	methyltransferase, putative, family protein [Mycobacterium avium 104]	5,00E-32	40%	ABK67089.1	**MAA104**
12	**MAP3486**	lactate 2-monooxygenase [Mycobacterium avium 104]	0	99%	ABK65670.1	**MAA104**
	**MAP3486**	LldD2 protein [Mycobacterium avium subsp. hominissuis TH135]	4,00E-35	32%	BAN31520.1	**MAH TH135**
13	**MAP3547c**	hypothetical protein MAV3388_23345 [Mycobacterium avium subsp. hominissuis 3388]	0	99%	KDO92449.1	**MAH**
	**MAP3547c**	conserved hypothetical protein [Mycobacterium avium 104]	2,00E-17	26%	ABK65078.1	**MAA104**
14	**MAP3680c**	Formate dehydrogenase [Mycobacterium avium subsp. hominissuis TH135]	0	99%	BAN33415.1	**MAH TH135**
	**MAP3680c**	formate dehydrogenase [Mycobacterium avium 104]	0	99%	ABK68013.1	**MAA104**
15	**MAP3731c**	O-antigen export system ATP-binding protein RfbB [Mycobacterium avium 104]	0,13	21%	ABK65477.1	**MAA104**
	**MAP3731c**	O-antigen export system ATP-binding protein RfbB [Mycobacterium avium subsp. hominissuis TH135]	0,16	21%	BAN29326.1	**MAH TH135**
16	**MAP3804**	glycosyl hydrolases family protein 16 [Mycobacterium avium 104]	0	100%	ABK67366.1	**MAA104**
	**MAP3804**	glycosyl hydrolases family protein 16 [Mycobacterium avium subsp. hominissuis TH135]	7,00E-174	100%	BAN33331.1	**MAH TH135**
17	**MAP4096**	nitroreductase family protein [Mycobacterium avium 104]	6,00E-115	99%	ABK64565.1	**MAA104**
	**MAP4096**	nitroreductase [Mycobacterium avium subsp. hominissuis TH135]	2,00E-04	23%	BAN29848.1	**MAH TH135**
18	**MAP4308c**	fructose-bisphosphate aldolase class-I [Mycobacterium avium 104]	0	99%	ABK68800.1	**MAA104**
19	**Ag5**	hypothetical protein MAPs_16490 [Mycobacterium avium subsp. paratuberculosis S397]	2,00E-166	100%	EGO37081.1	**MAP**
20	**Ag6**	MULTISPECIES: ATP-dependent helicase [Mycobacterium avium complex (MAC)	0,047	39%	WP_038536272.1	**MAC**
21	**MAP1637c**	M. avium UbD family decarboxylase	0,00E+00	99%	WP_016706304.1	***M*. *avium***
22	**MAP0388**	hypothetical protein MAP_0388 [Mycobacterium avium subsp. paratuberculosis K-10]	0	99%	AAS02705.1	**MAP**
23	**MAP3743-Ag9**	hypothetical protein MAP_3743 [Mycobacterium avium subsp. paratuberculosis K-10]	0	99%	AAS06293.1	**MAP**
24	**MAP3744m-Ag10**	thiazolinyl imide reductase [Mycobacterium avium subsp. paratuberculosis 10–4404]	0	99%	ETB06990.1	**MAP**
24	**MAP3744m-Ag10**	thiazolinyl imide reductase [Mycobacterium avium]	0	99%	WP_016705779.1	***M*. *avium***
25	**MAP0217**	antigen 85-C [Mycobacterium avium 104]	7,00E-180	99%	ABK64781.1	**MAA104**
	**MAP0217**	antigen 85-C protein [Mycobacterium avium subsp. hominissuis TH135]	3,00E-179	99%	BAN29303.1	**MAH TH135**
26	**MAP0353**	glycerol kinase [Mycobacterium avium 104]	0	99%	ABK67587.1	**MAA104**
27	**MAP1166**	RecName: Full = Triosephosphate isomerase; Short = TIM; Short = TPI; AltName: Full = Triose-phosphate isomerase	0	99%	A0QHY3.1	**MAA104**
28	**MAP1362**	arginine biosynthesis bifunctional protein ArgJ [Mycobacterium avium 104]	9,00E-145	99%	ABK65024.1	**MAA104**
29	**MAP1803**	dienelactone hydrolase [Mycobacterium avium subsp. hominissuis TH135]	4,00E-166	99%	BAN31765.1	**MAH TH135**
	**MAP1803**	dienelactone hydrolase family protein [Mycobacterium avium 104]	2,00E-143	99%	ABK69268.1	**MAA104**
30	**MAP2800**	enoyl-CoA hydratase [Mycobacterium avium 104]	0	99%	ABK64508.1	**MAA104**
	**MAP2800**	enoyl-CoA hydratase [Mycobacterium avium subsp. hominissuis TH135]	2,00E-24	30%	BAN30137.1	**MAH TH135**
31	**MAP2864c**	dihydrodipicolinate synthase [Mycobacterium avium 104]	0	99%	ABK66322.1	**MAA104**
32	**MAP3413**	aldehyde dehydrogenase (NAD) family protein [Mycobacterium avium 104]	0	99%	ABK69290.1	**MAA104**
	**MAP3413**	P-cumic aldehyde dehydrogenase [Mycobacterium avium subsp. hominissuis TH135]	3,00E-41	27%	BAN31590.1	**MAH TH135**
33	**MAP3634**	ErfK/YbiS/YcfS/YnhG family protein [Mycobacterium avium 104]Lipoprotein lprQ	0	100%	ABK64614.1	**MAA104**
	**MAP3634**	hypothetical protein MAH_1464 [Mycobacterium avium subsp. hominissuis TH135]	6,00E-48	41%	BAN30538.1	**MAH TH135**
34	**MAP3668c**	phosphotriesterase-like protein [Mycobacterium avium subsp. hominissuis TH135]	0	99%	BAN33426.1	**MAH TH135**
	**MAP3668c**	phosphotriesterase-like protein [Mycobacterium avium 104]	0	99%	ABK64738.1	**MAA104**
35	**MAP2541c**	Malate dehydrogenase	0	99%	A0QCI6.1	**MAA104**

Ags 1–18: *Map* antigens identified by proteomic and immunoproteomic analysis of *Map* culture filtrate [26]; Ags 19–24: *Map* antigens identified by *in silico* predictions [27]; Ags 25–35: *Map* antigens identified in *Map* cultures submitted to stress conditions: nutrient starvation, hypoxia or acidic pH (Roupie *et al*., manuscript in preparation). Proteins actually upregulated in stress conditions are highlighted in grey. Putative function and results of BlastP comparison (E value and % protein identity) with *Mah*104 and *Mah* TH135 genome.

### Antibody responses

Pooled sera of five mice infected by the porcine MST**12** isolate were collected at week 20 post-infection and analyzed for mycobacteria-specific IgG antibodies against whole *Map* culture filtrate by ELISA and against the 35 recombinant *Map* proteins (100 ng/well in borate buffer) using a dot-blot coupled to Western blot using pooled sera/group diluted 1:50, peroxidase-labelled rat anti-mouse IgG (LO-MK-1, purchased at Experimental Immunology Unit, Université Catholique de Louvain, Brussels, Belgium) diluted 1:500 and α-chloronaphtol (Sigma-Aldrich, St. Louis, MO, USA) in H_2_O_2_ for revelation. Reaction was stopped by washing in tap water. As the dot-blot assay required serum volumes in the order of 0.5 mL, sera were pooled. Non-infected mice were used as negative control.

### Statistical analysis

GraphPad Prism software (San Diego, CA, USA) was used to perform statistical analysis. One-way ANOVA (with Tukey’s post-t-tests) and Student t tests were performed to demonstrate statistical differences. For all tests p-values < 0.05 were considered significant.

## Results

### Bacterial replication of 8 *Mah* isolates in intranasally infected BALB/c mice

**Fig 1:**
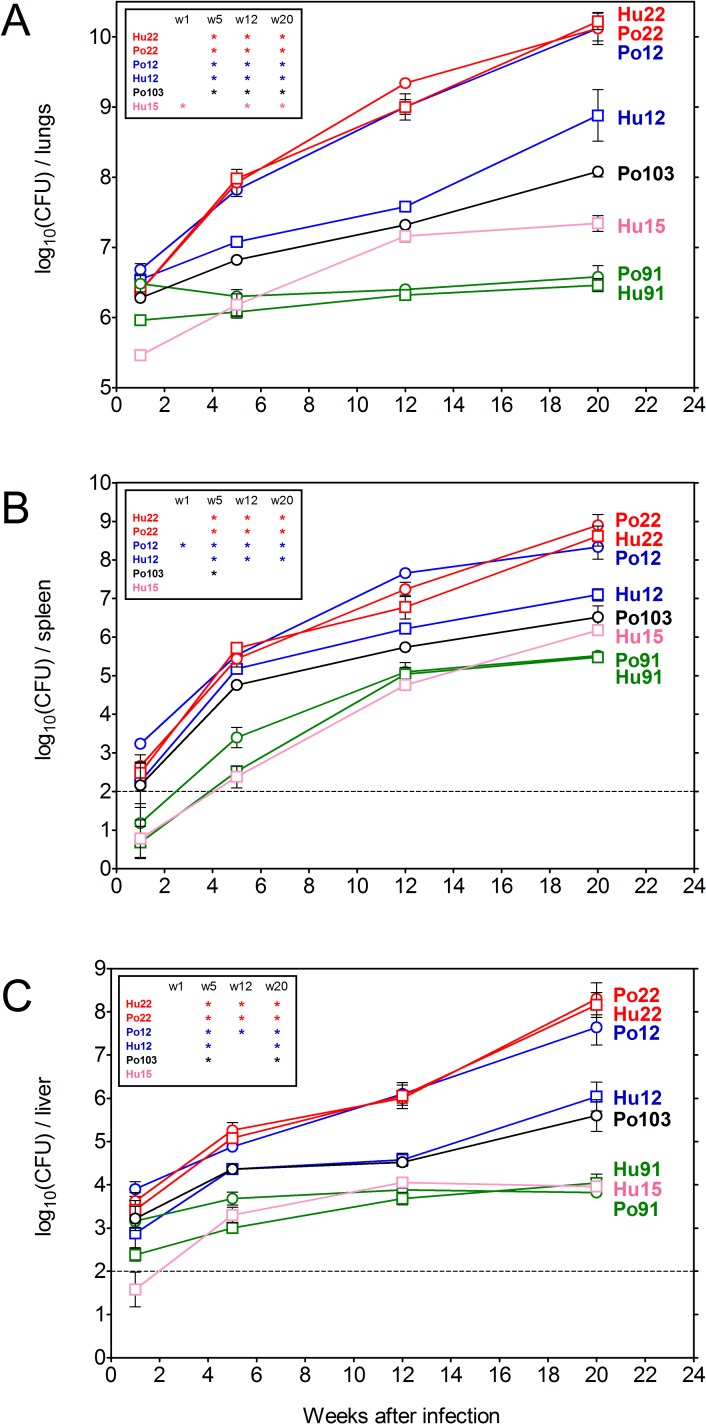
**Bacterial replication in lungs (A), spleen (B) and liver (C) of BALB/c mice infected with one of the eight *Mah* isolates, as measured at 1, 5, 12 and 20 weeks post-infection.** Data represent mean CFU (log_10_)/organ ± SEM of five animals tested per group. Isolate IDs represent the host (Hu: human; Po: swine) followed by the respective multispacer sequence type (MST). Statistical significance is depicted for each strain as compared to both Po**91** and Hu**91** (*: p<0.05).

BALB/c mice were infected by the intranasal route with 10^7^ CFU of the eight *Mah* isolates listed in Table 1. Virulence of the strains was examined, by enumeration of the bacteria in lungs, spleen and liver at week 1, 5, 12 and 20, as previously reported for testing *Map* virulence [18]. Pronounced differences were observed in bacterial replication of the eight isolates and their MST type seemed to correlate with this variation for four of them (Fig 1). Thus, the human and porcine isolates with shared MST**22** (in red) showed a more than 1,000 fold increase in bacterial numbers in lungs (6.5 log_10_ at week 1 to 10 log_10_ at week 20) and a dramatic dissemination to spleen (2 log_10_ at week 1 to 8 log_10_ at week 20) and liver (3.5 log_10_ at week 1 to 8 log_10_ at week 20), whereas the human and porcine isolates with shared MST**91** (in green) showed no replication in lungs (stable CFU counts at 6.5 log_10_ throughout the entire period) or liver (stable CFU counts at 3 log_10_) and minor dissemination to spleen (1.0 log_10_ to 3 log_10_). For the two MST**12** isolates, the link with bacterial replication was less clear as the porcine MST**12** isolate replicated to the same extent as the two MST**22** isolates, whereas the human MST**12** isolate showed intermediate virulence. Porcine MST**103** finally also showed an intermediate replication and human MST**15** isolate finally replicated to a minor extent in the lungs, but did not disseminate to liver or spleen.

Another infection run was realized with seven of these eight *Mah* strains. The results of this run (lacking the Hu**15** isolate, and with time-point measures at 5, 9 and 20 weeks post-infection) are shown in S1 Fig. A similar variation in bacterial replication, associated with a thousand fold increase in bacterial numbers in lungs of mice infected with the two MST**22** isolates and the porcine MST**12** isolate and a stable number of bacteria in the lungs of mice infected with the two MST**91** isolates was observed.

### Cytokine production

**Fig 2:**
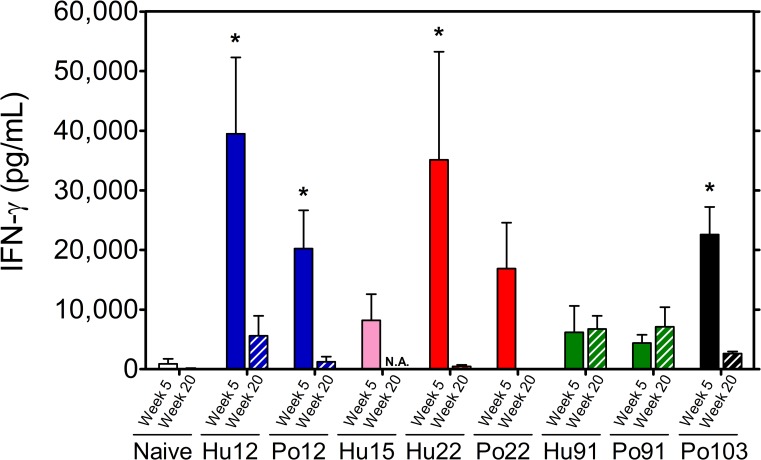
Spleen cell IFN-γ production in response to *M*. *avium* culture filtrate (5 μg/mL) in BALB/c mice infected for 5 weeks (filled bars) or 20 weeks (striped bars) with the different *Mah* isolates. Results represent mean IFN-γ levels (pg/mL) ± SEM of 3–5 mice tested individually. Statistical significance is depicted for each human or porcine strain as compared to respectively Hu**91** or Po**91** (*: p<0.05). N.A.: not available.

**Fig 3:**
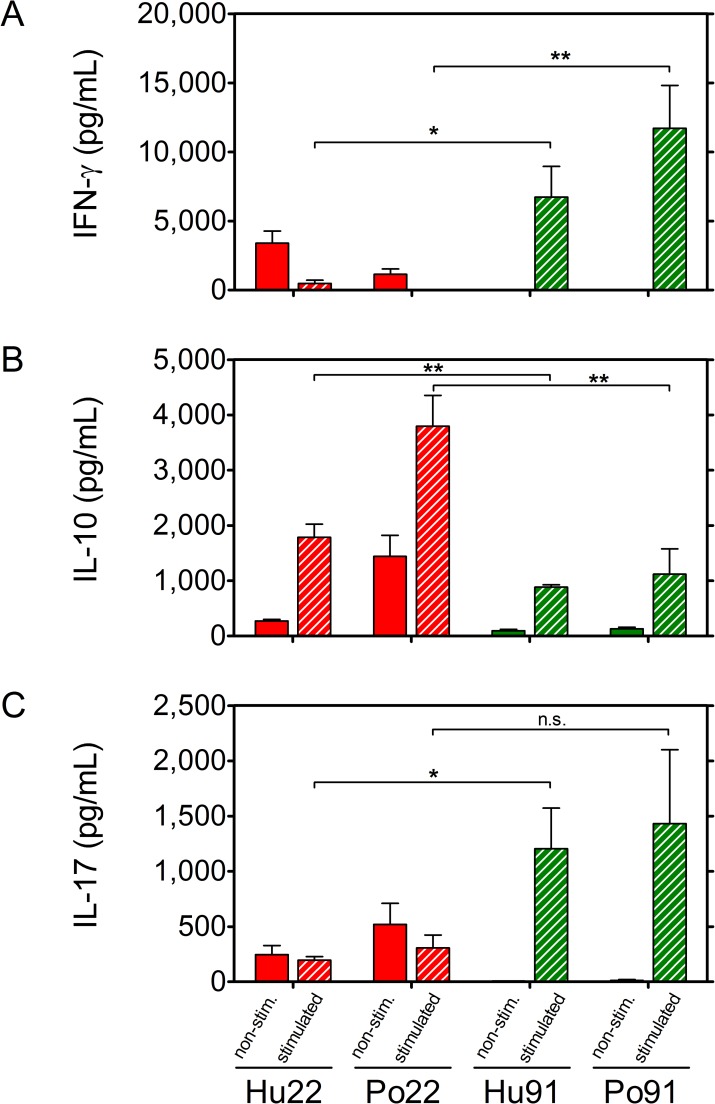
**IFN-γ (A), IL-10 (B) and IL-17 (C) production in 72h spleen cell culture supernatant of mice infected for 20 weeks with human and porcine MST22 (red) and human or porcine MST91 (green) *Mah* isolates.** Cells were unstimulated (filled bars) or stimulated with culture filtrate (striped bars). Results represent mean cytokine levels (pg/mL) ± SEM of 5 mice tested individually in each group. Statistical significance is depicted for MST**22** strains as compared to MST**91** strains (*: p<0.05; **: p<0.005; n.s.: non-significant).

At five weeks post-infection, strong IFN-γ production was detected in response to *M*. *avium* culture filtrate (and PPD, data not shown) in mice infected with MST**12** and MST**22** isolates (ranging between 20,000 and 40,000 pg/mL), whereas IFN-γ levels were about tenfold lower in mice infected with MST**91** isolates (Fig 2). At week 20 p.i., IFN-γ responses induced by *M*. *avium* CF were completely abrogated in mice infected with the human and porcine MST**22** and the porcine MST**12** isolates, whereas IFN-γ responses in mice infected with both *Mah* MST**91** isolates were sustained at levels even slightly, although not significantly higher than at the early time point (Figs 2 and 3A).

In order to find out, whether the suppressed IFN-γ response at 20 weeks post-infection in spleen of MST**22** infected mice was the result of a shift from Th1 to Th2, Th17 or Treg helper T cell phenotype, we measured the production of IL-4, IL-10, IL-17 and IL-1β by ELISA in week 5 and week 20 spleen cell culture supernatants of mice infected with the two MST**22** and the two MST**91** isolates. At week 5, only weak IL-10 levels were detected in culture supernatants of the four groups (data not shown), but at week 20 a significantly higher IL-10 production was measured in supernatants of MST**22** than of MST**91** infected mice (p<0.005) (Fig 3B), suggesting that the abrogated IFN-γ response in the MST**22** infected mice may have been the result of an activation of IL-10 producing regulatory macrophages and/or T cells. IL-17 could be detected only in week 20 culture supernatants (Fig 3C). In MST**22** infected mice, levels of IL-17 were comparable in supernatants from non-stimulated or CF-A stimulated cells, whereas in MST**91** infected mice, stimulation with CF-A induced increased IL-17 production as compared to non-stimulated cells. IL-4 levels were below detection level at the two time points for all four *Mah* groups, and very weak IL-1β levels were detected that were not different between the two time points and the four *Mah* groups (data not shown).

### IFN-γ production and IgG antibodies in response to 35 recombinant, purified *Map* antigens in *Mah* infected BALB/c mice

**Fig 4:**
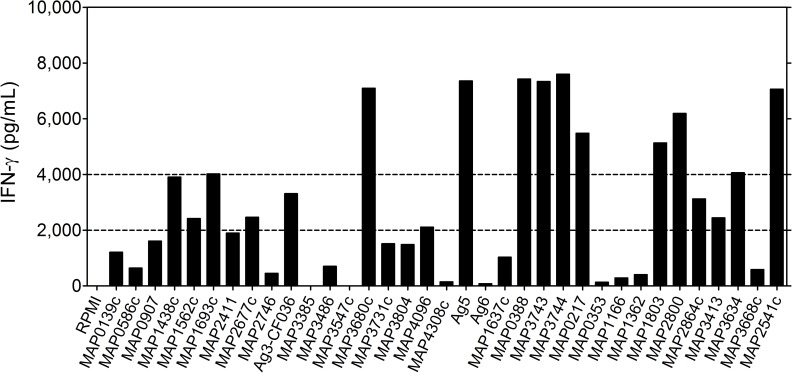
IFN-γ response of BALB/c mice infected intranasally with porcine MST12 isolate (Po12). Mice (n = 5) were sacrificed 5 weeks post-infection and pooled spleen cells were stimulated for 72h with each of the 35 *Map* antigens (5 μg/mL). IFN-γ levels are expressed in pg/mL.

A series of 35 *M*. *avium* subsp. *paratuberculosis* protein sequences were compared for identity with proteins in *Mah* 104 and *Mah* TH135, using BlastP (Table 2). Except for the six *in silico* predicted antigens (antigens 19–24 in Table 2), predicted identity between the *Map* and *Mah* proteins was in the order of 99% for twenty-six of the twenty-nine other proteins (Table 2). Three MAP proteins, i.e. Ag 3, MAP3385 and MAP3731, showed a lower percentage of identity with *Mah* of 20%, 64% and 21% respectively. In view of these strong identities and as cloned and purified *Mah* products were not available, we tested the purified *Map* proteins in an IFN-γ release assay (Fig 4). Nine of the thirty-five antigens induced strong IFN-γ responses (> 4,000 pg/mL) at week 5 p.i. in spleen cell cultures from mice infected with virulent porcine MST**12**: MAP3680c a formate dehydrogenase, MAP0217 (Ag85C) a member of the highly conserved immunodominant Ag85 family, which functions as mycolyl-transferase involved in the coupling of trehalose to the arabinogalactan of the mycobacterial cell wall [29], MAP1803 belonging to the dienelactone hydrolase family protein, MAP2800 predicted to be an enoyl-CoA hydratase and MAP2541 finally a malate dehydrogenase. Somewhat surprisingly, the hypothetical proteins Ag5, MAP0388, MAP3743 and the predicted thiazolinyl imide reductase MAP3744, all four previously selected *in silico* (and for which a new blast analysis confirmed the absence of homology with other *M*. *avium* subspecies) also stimulated a strong IFN-γ production in spleen cells of mice infected with the MST**12** *Mah* isolate.

**Fig 5:**
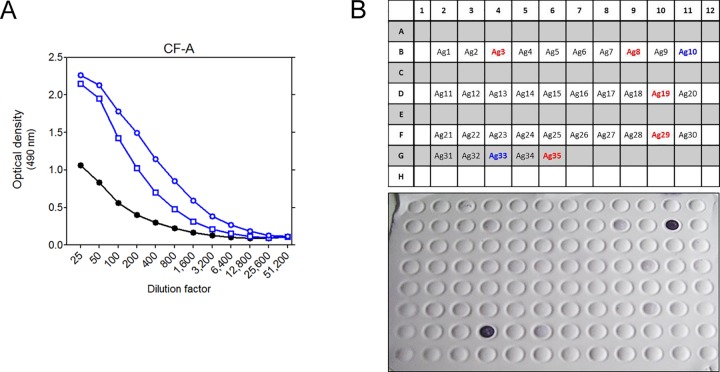
A: IgG antibodies against *M*. *avium* culture filtrate in serial dilutions of pooled sera of BALB/c mice infected for 20 weeks with porcine (blue circles) or human (blue squares) *Mah* MST12 isolate (pooled sera from naïve mice represented by black dots). B: Plate organization and Western blot image of immunoblot of week 20 pooled *Mah* Po12 serum tested against the 35 *Map* antigens. Numbers of the two antigens giving the strongest signals are indicated in blue, numbers of the five antigens giving weaker signals are indicated in red.

Serial dilutions of pooled sera of BALB/c mice infected for 20 weeks with porcine Po**12** and human Hu**12** or sera from non-infected mice were tested against *M*. *avium* CF by ELISA (Fig 5A). Strong CF-A specific antibodies were detected in sera from *Mah* Po**12** and Hu**12** infected mice. Pooled sera from *Mah* Po**12** infected mice were also tested against the 35 *Map* antigens in a dot-blot assay (Fig 5B). Two proteins were strongly recognized (Nr.10/CF-036 and Nr.33/MAP3634, homologous to lprQ lipoprotein) and five proteins (Nr.3/MAP0907, a predicted morphine 6-dehydrogenase, Nr.8/MAP2677c of the glyoxalase family protein, Nr.19/Ag5, Nr.29/MAP1803 and Nr.35/MAP2541c) were recognized but more weakly in the dot-blot. These results are consistent with other dot-blot experiments in which MAP3634 was also very strongly recognized by sera from *M*. *avium* subsp. *avium* infected mice (data not shown), suggesting this antigen had a potential for serodiagnosis.

### Comparison of sequences of MST22 and MST91 isolates with sequences of two *Mah* reference strains

As differences in MST type seemed to be reflected (at least to some extent) by differences in virulence in BALB/c mice, whole genome sequencing of the two MST**22** and the two MST**91** isolates was performed, sequences have been deposited at the European Nucleotide Archive and genome statistics of the four isolates was reported [19]. MST**22** rather than MST**12** isolates were chosen for sequencing because of the limited clinical information for the human MST**12** isolate.

Next, the genome sequences of MST**22** and MST**91** isolates were compared with two *M*. *avium* subsp. *hominissuis* reference strains, i.e. *Mah* 104 and *Mah* TH135, isolated from an AIDS patient with disseminated disease and a HIV-negative patient with pulmonary disease respectively. In a very interesting study, the genome of these two reference strains was compared by Uchiya *et al*. and described to encode for a series of specific virulence factors [21]. Here, we analyzed whether the sequences of these virulence factors were also present in the genome of the MST**22** and MST**91** isolates (Table 3). One gene sequence from the *Mah* TH135 reference strain, namely MAH_1657, and ten other sequences from the *Mah* 104 reference strain were found to be present at 100% identity in both MST**22** isolates (Hu**22** and Po**22**). For the MST**91** isolates, only two gene sequences (MAV_0117 and MAV_0953) were found to be identical with the sequence of the genes reported from the reference strain *Mah* 104. Interestingly, the nine gene sequences present in the two virulent isolates of genotype **22** and coding for specific virulence factors of the mammalian cell entry (Mce family) are indeed absent in the two avirulent isolates of genotype **91**. Among these nine genes, five were localized by Uchiya *et al*. in a SR21 strain-specific region identified in the *Mah* 104 reference strain [21]. Besides the *Mce* genes, two genes identical to PPE proteins were found in MST**22** isolates: MAH_1657 (TH135) sharing 100% identity and MAV _1347 (104) 99% identical with *M*. *avium* subsp. *avium*. Genomes of the two MST**91** isolates had an identical copy MAV_0117, encoding a PE protein, 99% identical with *M*. *avium* subsp. *avium*. Finally, BlastN analysis did not reveal any sequence homology in our four Belgian isolates with the plasmid pMAH135 (data not shown), which was reported to be associated with progression to pulmonary disease in Japan [30].

**Table 3:** Genes present at 100% identity in the sequenced *Mah* strains.

**Genes identified by [21] in reference strain *Mah* TH135**					
Gene sequence name	Predicted protein	Presence in Hu**22**	Presence in Po**22**	Presence in Hu**91**	Presence in Po**91**
MAH_1657	PPE	X	X		
**Genes identified by [21] in reference strain *Mah* 104**					
Gene sequence name	Predicted protein	Presence in Hu**22**	Presence in Po**22**	Presence in Hu**91**	Presence in Po**91**
MAV_RS00575/MAV_0117	PE			X	X
MAV_RS04540/MAV_0948	Mce	X	X		
MAV_RS04550/MAV_0950	Mce	X	X		
MAV_RS04555/MAV_0951	Mce	X	X		
MAV_RS04565/MAV_0953	Mce			X	X
MAV_RS06460/MAV_1347	PPE	X	X		
MAV_RS24220/MAV_5047	SR-21 Mce	X	X		
MAV_RS24225/MAV_5048	SR-21 Mce	X	X		
MAV_RS24230/MAV_5049	SR-21 Mce	X	X		
MAV_RS24240/MAV_5051	SR-21 Mce	X	X		
MAV_RS24245/MAV_5052	SR-21 Mce	X	X		

## Discussion

A worldwide increase in the prevalence of human nontuberculous mycobacterial (NTM) infections has been observed since 2000 [31]. NTM infections are thought to be triggered essentially by exposure to environmental NTM that reside in soil and water. In recent years it has become clear that members of the *Mycobacterium avium* complex such as the subspecies *M*. *avium* subsp. *hominissuis* (*Mah*) cause a problem in immunocompetent adults (mostly respiratory tract infections) and children (lymphadenitis) as well as in immunocompromised AIDS patients [32,33]. Interestingly, advancement in sequence-based identification and genotyping has demonstrated a remarkable genetic diversity of the *Mah* subspecies [2].

On the basis of multisequence typing, we selected four swine and four human *Mah* isolates for a detailed analysis of *in vivo* virulence and confirmed a strong variation between the different isolates. Monitoring bacterial replication in the lungs over a period of 20 weeks, a human and porcine isolate with MST**22** type were found to be highly virulent, whereas a human and porcine isolate with MST**91** type were found to be fully avirulent. Mycobacteria-specific IFN-γ responses were highest in mice infected with the virulent isolates early during infection, but at the late stage, these responses were markedly suppressed and in parallel IL-10 responses were increased. These findings are reminiscent of the report of Roque *et al*. who compared the susceptibility of BALB/c and C57BL/6 mice to intravenous infection with *M*. *avium* subsp. *avium* strain 2447 and showed that the higher susceptibility of BALB/c than of C57BL/6 mice was related to higher IL-10 responses in the former strain [34]. In contrast, mice infected with *Mah* MST**91** isolates, showed more modest but sustained IFN-γ levels throughout the entire study period, and at the late time point significant mycobacteria-specific IL-17 production could be measured, reflecting the presence of a possibly protective Th17 memory T cell population in mice infected with these *Mah* isolates of which the multiplication was controlled. Also in NTM patients with nodular bronchiectasis, it was suggested that susceptibility to pulmonary disease may reflect low IL-17 and high IL-10 responses, rather than a Th1 deficiency [35].

Immunodiagnosis of *M*. *avium* infection is hampered by the lack of specific antigens. Avian and bovine tuberculins (which are purified protein derivatives of autoclaved bacteria) are the reagents commercially available to assess exposure to *Map* in cell-mediated immune assays such as intradermal skin tests and IFN-γ release assays, but these assays are hampered by the presence in PPDs of many cross-reactive antigens. The use of *M*. *avium* specific antigens may help to overcome this specificity issue if a sufficient sensitivity could be obtained. By screening a series of 35 antigens, many predicted to be specific for *M*. *avium*, we identified here some protein candidates that hold promise for the detection of early infection using IFN-γ release assays (CF-036 and MAP2541c) and for the serodiagnosis of multibacillary disease (MAP3634). The MAP3634 antigen (new name MAP_RS18645) has a predicted function of a lipoprotein-anchoring transpeptidase, also known as lipoprotein LprQ. Interestingly, other lipoproteins such as the phosphate-binding protein PstS-1 of *M*. *tuberculosis* [36] and PstS-2 and PstS-3 of *M*. *bovis* BCG [37,38] are known to be powerful B cell antigens. A detailed analysis of the immunodiagnostic potential of these 35 proteins in cattle is in progress (Roupie *et al*., manuscript in preparation).

As stated by Winthrop *et al*., “studies of NTM pathogenesis and host immunity to NTM have been hampered by the lack of a robust animal model that can mimic human non-disseminated MAC pulmonary disease” [39]. Studies in non-human primates such as rhesus macaques could be a solution, but our results show that an intranasal infection of BALB/c mice with a properly selected avirulent strain such as the *Mah* MST**91** could be a valuable alternative.

Uchiya *et al*. reported on genetic diversity in *Mah* strains that cause pulmonary and disseminated disease, and performed a comparative genome analysis of *Mah*TH135, isolated from a HIV-negative patient with pulmonary disease [21] and *Mah*104, isolated from an adult AIDS patient in Southern California in 1983. The sequenced genome of *M*. *avium* strain 104 has been associated with disease in multiple patients in the western United States. Although *M*. *avium* is known for its genetic plasticity, this observation indicates that certain strains of the pathogen such as the *Mah* 104 isolate can be genotypically stable over extended periods of time [20]. In our study, the virulent human *Mah* MST**22** strain was isolated from a 40 year old HIV-negative man with disseminated disease and the avirulent human *Mah* MST**91** strain from a 40 year old AIDS patient with disseminated disease. Whole genome sequencing of these two human isolates was performed (in parallel with the MST**22** and MST**91** pig isolates) [19] and sequences were compared to the *Mah* TH135 and *Mah* 104 sequence, with respect to twenty-five candidate strain 104 specific genes and to twenty-two candidate strain TH135 specific genes, reported by Uchiya [21]. Porcine and human sequences aligned according to their MST type, and the two MST**22** isolates had ten identical *Mah* strain 104 specific genes and one *Mah* TH135 gene, whereas the two MST**91** isolates had only 2 genes identical with *Mah* strain 104 (Table 3). Among the nine putative Mce proteins identical in the MST**22** isolates, five are localized in the strain-specific SR-21 region of the reference 104 strain, region with a sequence similarity of 70–75% with *M*. *abscessus* [21]. Although the precise mechanisms involving Mce proteins in virulence remain unclear, these proteins are thought to help mycobacteria to enter into macrophages, but they have also been implicated in uptake of cholesterol essential for long term survival in host cells.

Thus, genomic comparison indicated that the human and porcine MST**22** isolates, were much more similar to the reference *Mah* 104 type than to the *Mah* TH135 type with respect to predicted virulence factors. *Mah* isolates exhibit geographic differences in genetic diversity, with isolates from Japan (such as *Mah* TH135) sharing a high degree of relatedness with Korean isolates, but not with isolates from Europe or the United States [2]. The strong genetic similarity of the human MST**22** strain isolated in 2012 in Belgium (from an HIV-negative patient) with the *Mah* 104 strain (isolated in 1983 in the USA from an AIDS patient) seems to indicate that genetic differences are determined by the geographic origin rather than by the type of clinical symptoms. On the other hand, Moriyama *et al*. reported recently on an association between the presence of a pMAH135 plasmid in *Mah* isolates and the progression of pulmonary disease [30]. Interestingly, although a plasmid seems indeed to be involved for Japanese isolates, in our study, careful genome comparison failed to find any homology with the pMAH135 sequence in the four Belgian isolates.

In conclusion, our results show that intranasal infection of BALB/c mice can be used as a reliable experimental model to analyse variations in virulence of different *Mah* isolates. Very recently, Uchiya *et al*. performed a comparative genome analysis of 79 *M*. *avium* strains and on the basis of single nucleotide polymorphisms a phylogenetic tree was drawn [40]. The four Belgian *Mah* isolates were included in the study and they localized on the phylogenetic tree two by two according to their MST genotype, more specifically in the IIa subcluster [40]. Therefore, the striking link of virulence and identity at the genome level of porcine and human isolates with the same multisequence type, for which no correlation of place of residence (humans) or farm of origin (pigs) was observed, points to the presence in the environment of certain genotypes of *Mah* which may be more infectious for both humans and pigs than other genotypes.

## Supporting information

S1 Fig **Bacterial replication in lungs (A), spleen (B) and liver (C) of BALB/c mice infected with one of the seven *Mah* isolates, as measured at 2, 5, 9 and 20 weeks post-infection.** Data represent mean CFU (log_10_)/organ ± SEM of five animals tested per group. Isolate IDs represent the host (Hu: human; Po: swine) followed by the respective multispacer sequence type (MST). Statistical significance is depicted for each strain as compared to both Po**91** and Hu**91** (*: p<0.05).
(TIF)

## Abbreviations

CODA-CERVA: Centrum voor Onderzoek in Diergeneeskunde en Agrochemie- Onderzoek- Centre d’Etude et de Recherches Vétérinaires et Agrochimiques
CODA-PIB-WIV: Centrum voor Onderzoek in Diergeneeskunde en Agrochemie-Pasteur Instituut van Brussel-Wetenschappelijk Instituut voor Volksgezondheid
IFN-γ: interferon-gamma
IL: interleukin
IgG: Immunoglobulin
MAC: *Mycobacterium avium* complex
MST: multispacer sequence type
Mce: mammalian cell entry
ni-NTA: nickel- nitrilotriacetic acid
NO: nitric oxide
NTM: non tuberculous mycobacteria
OADC: oleic acid-albumin-dextrose-catalase
OPD: o-phenylenediamine
PPD: purified protein derivative (of tuberculin)
CF-A: culture filtrate of *M*. *avium*
PPE: proline-proline-glutamic acid
PE: proline-glutamic acid
SR-21: Specific Region 21
WIV-ISP: Wetenschappelijk Instituut voor Volksgezondheid-Institut de Santé Publique
