# Supplementary figures and images for: Virulence and immunogenicity of genetically defined human and porcine isolates of M. avium subsp. hominissuis in an experimental mouse infection
Source: PLoS One. 2017 Feb 9;12(2):e0171895. doi: 10.1371/journal.pone.0171895 (PMC5300754; doi:10.1371/journal.pone.0171895)

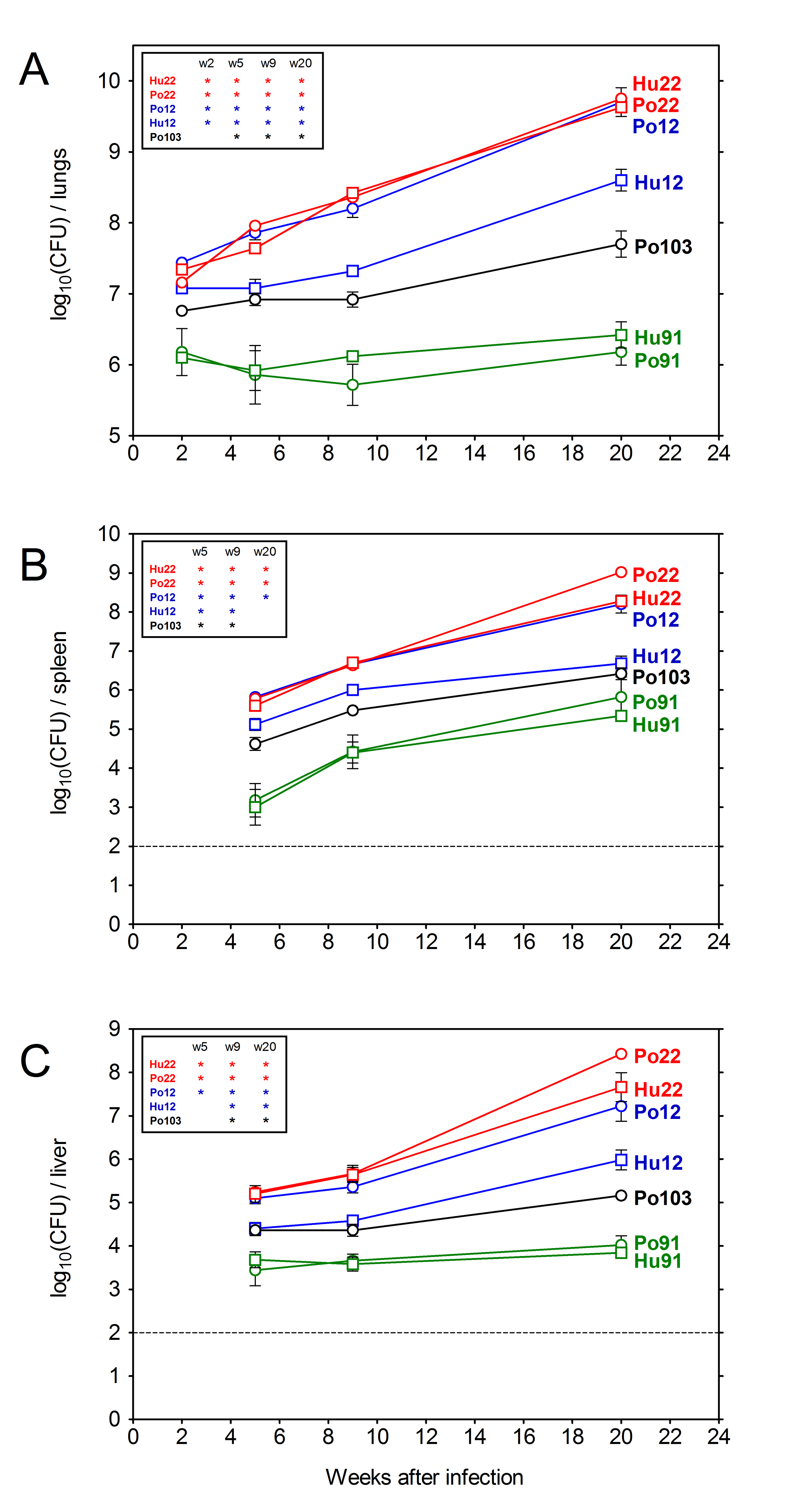

Supplement: S1 Fig — Bacterial replication in lungs (A), spleen (B) and liver (C) of BALB/c mice infected with one of the seven Mah isolates, as measured at 2, 5, 9 and 20 weeks post-infection. Data represent mean CFU (log10)/organ ± SEM of five animals tested per group. Isolate IDs represent the host (Hu: human; Po: swine) followed by the respective multispacer sequence type (MST). Statistical significance is depicted for each strain as compared to both Po91 and Hu91 (*: p<0.05). (TIF) [file pone.0171895.s001.tif]
